# Beneficial effect of resveratrol on phenotypic features and activity of osteoarthritic osteoblasts

**DOI:** 10.1186/s13075-017-1365-2

**Published:** 2017-06-30

**Authors:** Élie Abed, Aline Delalandre, Daniel Lajeunesse

**Affiliations:** Unité de recherche en Arthrose, Centre de recherche du Centre hospitalier de l’Université de Montréal (CRCHUM), 900, rue Saint-Denis, Montréal, Québec H2X 0A9 Canada

**Keywords:** Osteoarthritis, Subchondral bone, Canonical Wnt signaling, Resveratrol, Mineralization, Sirtuin 1

## Abstract

**Background:**

Osteoarthritis (OA) is a complex disease, which affects multiple tissues, namely the subchondral bone, articular cartilage and synovial membrane. Alterations of the subchondral bone include an increased, yet under mineralized osteoid matrix, abnormal osteoblast cell phenotype including elevated alkaline phosphatase (ALP) activity, increased release of osteocalcin (OC) and transforming growth factor β-1 (TGF-β1). Previous studies have demonstrated an inhibition of the canonical Wnt signaling (cWnt) pathway in OA osteoblasts (Ob). As resveratrol (RSV) has been shown to upregulate the Wnt signaling pathway in different cell systems, we hypothesized that RSV could be beneficial for OA Ob.

**Method:**

We prepared primary human Ob using the subchondral bone plate of tibial plateaus of OA patients undergoing total knee arthroplasty, or tibial plateaus of normal individuals at autopsy. Sirtuin 1 (Sirt1) expression in normal and OA subchondral bone tissue was evaluated by immunohistochemical analysis. Expression of genes was evaluated by qRT-PCR and protein production by western blot analysis. ALP activity and osteocalcin secretion were evaluated respectively with substrate hydrolysis and enzyme immunoassay. Mineralization levels were evaluated with alizarin red staining. Wnt/β-catenin signaling was evaluated by target gene expression using the TOPflash TCF/lef luciferase reporter assay and intracellular signaling using β-catenin levels in western blot analysis. Extracellular signal-regulated kinase (Erk)1/2 and the Smad1/5/8 pathways were evaluated by western blot analysis.

**Results:**

Sirt1 expression and production were reduced in OA subchondral bone tissue compared to normal tissue. RSV upregulated Sirt1 and its activity, and reduced the expression of leptin. RSV increased Erk1/2 phosphorylation in OA Ob; however, it had no effect on Smad 1/5/8 phosphorylation. RSV had little effect on cell proliferation and only slightly affected the Bax/Bcl2 ratio. The expression of Runx2/Cbfa1 and peroxisome proliferator-activated receptor (PPAR)γ were not affected by increasing doses of RSV. The endogenous increased ALP activity and OC release observed in OA Ob compared to normal Ob were partly corrected only for ALP at high RSV levels but not for OC release. In contrast, RSV increased the mineralization of OA Ob. Moreover, whereas Wnt3a stimulates the Wnt/β-catenin pathway in these cells, RSV further increased the response to Wnt3a.

**Conclusion:**

These data indicate that RSV promotes Sirt1 levels, inhibits the endogenous expression of leptin by OA osteoblasts and can promote the Wnt/β-catenin and Erk1/2 signaling pathways, which are altered in these cells.

## Background

The exact mechanism that leads to osteoarthritis (OA) remains unknown; however, recent studies indicate that the subchondral bone tissue is implicated in the progression and/or the initiation of OA [1]. Cartilage damage, loss and failure to repair damage are characteristics of OA. It was believed this was restricted to abnormal chondrocyte function, yet recent studies using both clinical and animal models have underlined the crucial role played by the subchondral bone tissue in this process. Indeed, subchondral bone tissue is abnormal in OA patients and osteoblasts (Ob) from OA subchondral bone have altered functions [2, 3]. The Wnt signaling pathway is crucial for normal skeletal tissue homeostasis and function. In OA patients, the subchondral bone tissue is altered [4–6] and we previously showed that OA subchondral Ob have altered functions [2, 7, 8]. Moreover, we reported that the abnormal expression of phenotypic markers and reduced mineralization of OA Ob are linked with stimulation of the Wnt antagonist dickkopft-2 (DKK2) [9] and sclerostin (SOST) [10], and inhibition of the Wnt agonist, R-spondin 2 (Rspo2) [11].

Resveratrol (3,4′,5-trihydroxystlben (RSV)) is a natural product found in most grape cultivars. Resveratrol is recognized as a major phytoestrogen and has been shown to possess estrogenic activity [12]. Although the effect of RSV was mostly demonstrated in endothelial cells where it alters endothelial activities [13], recent studies have revealed its potential role in Ob. Indeed, RSV was shown to promote Ob proliferation and differentiation of multipotent mesenchymal cells [14] and to enhance the proliferation and differentiation of osteoblastic MC3T3-E1 cells [15] in a mouse cell model of osteoblasts. In contrast, RSV can also suppress the proliferation of osteosarcoma cells via a role in cell apoptosis [16]. Recent studies further demonstrated that RSV plays its role via its modulation of the Wnt/β-catenin signaling pathway by promoting Ob differentiation of multipotent mesenchymal cells [17]. The activation of the Wnt/β-catenin pathway by RSV triggers other signaling pathways such as extracellular signal-regulated kinase (Erk)1/2 in multipotent mesenchymal cells [17], and of note, the activation of the Erk1/2 pathway is responsible for the differentiation of mesenchymal cells into Ob [18].

Osteoarthritic Ob present a number of altered phenotypic features among which increased alkaline phosphatase (ALP) activity, osteocalcin release, and type 1 collagen expression, and reduced in vitro mineralization are but a few examples of these alterations [2, 3, 7, 9, 19]. Recent evidence indicates that alterations in the Wnt/β-catenin signaling pathway are responsible, at least in part, for the alterations of phenotypic features and reduced mineralization observed in OA Ob [7]. Indeed, Wnt/β-catenin activity is reduced in Ob in OA due to elevated levels of the Wnt antagonist DKK2 [9], elevated production of SOST [10], yet another Wnt antagonist, and reduced levels of the Wnt agonist Rspo2 [11].

Osteoarthritic Ob also have altered responses to insulin-like growth factor 1 and leptin treatments in part due to altered Erk1/2 and Smad1/5/8 signaling [20]. Of note, under hypoxic conditions, a situation observed in OA patients [21–24], leptin and DKK2 are further upregulated in these cells compared to cells under normorxic conditions [25]. In vitro mineralization is also altered in osteoblasts in OA [2] in response to alterations in transforming growth factor-β1 (TGF-β1) levels and reduced Wnt/β-catenin signaling activity [9]. In addition, Ob express more hepatocyte growth factor (HGF) than normal Ob in OA, and increased endogenous HGF production stimulates the expression of TGF-β1 and reduces the response to bone morphogenetic protein 2 (BMP-2) and mineralization in osteoblasts in OA [26].

As resveratrol has been shown to regulate the activity of Wnt/β-catenin in mesenchymal stem cells (MSC) cells [17], increase Erk1/2 and Akt, AMPK, Smad 1/5/8, p38, ERK,c-Jun N-terminal kinase, and enhance nuclear factor-κB activity in OM-stimulated cells [27], whereas some of these pathways are altered in Ob in OA, we questioned if RSV could correct these activities and which signaling pathways are involved in the potential response to RSV.

## Methods

### Patients and clinical parameters

Tibial plateaus were obtained from OA patients undergoing total knee replacement surgery and prepared as previously described [7, 19, 28]. A total of 41 patients (67.9 ± 7.8 years, mean ± SD; 15 male/26 female patients), who had OA according to the recognized clinical criteria of the American College of Rheumatology, were included [29]. No patients had received medication that would interfere with bone metabolism, including corticosteroids, for 6 months before surgery. A total of 12 subchondral bone specimens from normal individuals (age 64.3 ± 13.1 years, mean ± SD; 9 male/3 female individuals) were collected at autopsy within 12 h of death. They had not been on any medication that could interfere with bone metabolism or had any bone metabolic disease or abnormal cartilage macroscopic changes. All human samples were acquired following a signed agreement by the patients undergoing knee surgery and, for the specimens collected at autopsy, by the relatives of the deceased, in accordance with the ethics committee guidelines of the Centre de recherche du Centre Hospitalier de l’Université de Montréal (CRCHUM).

### Preparation of primary subchondral bone cell culture

Isolation of the subchondral bone plate and cell cultures were prepared as previously described using the medial tibial plateaus where bone sclerosis is observed [19]. At confluence, cells were passaged once at 25,000 cells/cm^2^ in 100-mm petri dishes and grown for 5 days in Ham F12/DMEM medium (Sigma-Aldrich, Oakville, Canada) containing 10% FBS. Confluent cells were then incubated in the presence or absence of 1,25(OH)_2_D_3_ (50 nM) for 48 h for the determination of biomarkers in the presence of 2% FBS. Supernatants were collected at the end of the incubation and kept at −80 °C prior to assay. Cells were prepared either in ALP buffer for phenotypic determinations or in TRIzol for RT-PCR experiments. Protein determination was by the bicinchoninic acid method [30]. Resveratrol was used at concentrations ranging from 10 nM to 1000 nM following previously published in vitro experiments [14–17], which reflect in vivo doses used in animal studies [13, 31]. Where indicated, phosphorylation of Erk1/2 was inhibited by the selective inhibitor PD98059 at a final concentration of 10 μM.

### Phenotypic characterization of human subchondral Ob cell cultures

ALP activity was determined by substrate hydrolysis using p-nitrophenylphosphate of whole cell lysates whereas osteocalcin release in cell supernatants was evaluated using an enzyme immunoassay (EIA) as previously described [7, 19]. Determinations were performed in duplicate for each preparation.

### Preparation of Wnt3a-conditioned medium (Wnt3a-CM)

Murine L cell lines transfected with either an empty vector (CRL-2648) or Wnt3a (CRL-2647) were obtained from the American Culture Type Collection (Cedarlane Laboratories Ltd, Burlington, ON, Canada). Control (L-CM) and Wnt3a-conditioned medium (Wnt3a-CM) was prepared using these cells. Briefly, the cells were grown in BGJb medium for 48 h after which conditioned medium (CM) was collected. CM was filtered sterilized, aliquoted and stored at –80 °C prior to use. CM was added to normal and OA Ob at a 10% final concentration.

### RT-PCR assays

For RT-PCR assays, total cellular RNA was extracted with the TRIzol^TM^ reagent (Invitrogen, Burlington, ON, Canada) according to the manufacturer’s specifications and treated with the DNA-free^TM^ Dnase Treatment and Removal kit (Ambion, Austin, TX, USA) to ensure complete removal of chromosomal DNA. The RNA was quantitated using the RiboGreen RNA quantification kit (Molecular Probes, Eugene, OR, USA). The RT reactions were primed with random hexamers with 1 ug of total RNA in a 100-μl final reaction volume followed by PCR amplification with the Rotor-Gene 6® RG-3000A (Corbett Research, Mortlake, NSW, Australia) as previously described [9, 10] using 20 pmol of each specific PCR primer. Gene specific primers were: ALP, F:ACGTGG CTAAGAATGTCATC, R: CTGGTAGGCGATGTCCTTA; osteocalcin (OC), F: ATGAGAGCCCTCACACTC, R: GAAAGCCGATGTGGTCAG; PPARG, F: TCTCTCCGTAATGGAAGACC, R: GCATTATGAGACATCCCCAC; LEPTIN, F: GGCTTTGGCCCTATCTTTTC, R: GGATAAGGTCAGGATGGGGT; Sirt1, F: GCTGGAACAGGTTGCGGGAA, R: GGGCACCTAGGACATCGAGGA; P300, F: GCAGTGTGCCAAACCAGATG, R: GGGTTTGCCGGGGTACAATA; glyceraldehyde-3-phosphate dehydrogenase (GAPDH), F: CAGAACATCATCCCTGCCTCT, R: GCTTGACAAAGTGGTCGTTGA G; RUNX2, F: AGATGATGACACTGCCACCTCTG, R: GGGATGAAATGCTTGGGAACTGC; these were added at a final concentration of 200 nM. The data were collected and processed with the GeneAmp 5700 SDS software and given as threshold cycle (Ct). Ct values were converted to number of molecules using standard curves for each target gene and values were expressed as the ratio of the number of molecules of the target gene to GAPDH.

### Evaluation of mineralization

Confluent cells were incubated in BGJb medium containing 10% FBS, 50 μg/ml ascorbic acid and 50 μg/ml β-glycerophosphate. This medium was changed every 2 days until day 28. Mineralization of cell cultures was evaluated by quantification of alizarin red staining performed following the extraction procedure of Gregory [32].

### Western immunoblotting

Cell extracts were loaded onto polyacrylamide gels and separated by sodium dodecyl sulfate-polyacrylamide gel electrophoresis (SDS-PAGE) under a reducing condition as previously described [2, 3]. Loading of the protein was adjusted according to the cellular protein concentration of each specimen. The proteins were then electrophoretically transferred onto nitrocellulose membranes (Boehringer Mannheim, Penzberg, Germany), and immunoblotting was performed as described in the ECL Plus western blotting detection system manual (Amersham Pharmacia Biotech, UK, England). We used rabbit anti-p300 at a dilution of 1:500 (Santa Cruz Biotechnology), rabbit anti-smad1/5/8 and rabbit anti-p-smad1/5/8 at a dilution of 1:1000 (Cell Signaling Technology, Beverly, MA, USA), rabbit anti-Sirt1 at a dilution of 1:1000 (Cell Signaling), rabbit anti-β-catenin and rabbit anti-phosphorylated β-catenin at dilutions of 1:2000 and 1:1000, respectively (Cell Signaling Technology), rabbit anti-p44/42 and rabbit anti-Phospho-p44/42 mitogen-activated protein kinase (MAPK) (Erk1/2) at dilutions of 1:1000 (Cell Signaling Technology), rabbit anti-Phospho-Smad1/5/8 at dilutions of 1:1000 (Cell Signaling Technology), and rabbit anti-human actin at a dilution of 1:10,000 (Sigma-Aldrich) as primary antibodies; a horseradish peroxidase (HRP)-conjugated goat anti-rabbi t IgG (1:10,000, Upstate Biotechnology, NY, USA) was used as the secondary antibody for the western blot assays. Densitometry analysis of western blot films was performed using the public domain National Institutes of Health (NIH) Image program developed with the Scion Image 1.63 program (Research Services Branch (RSB)). The public domain NIH Image program was developed at the US NIH (http://rsb.info.nih.gov/nih-image/).

### TOPflash dual-luciferase reporter assays

Primary normal and OA Ob were plated in 24-well plates at a density of 1.5 × 10^5^ cells/well containing 10% FBS in BGJb medium and left to recover overnight. Plasmid mixtures containing 2 μg TOPflash luciferase construct (Upstate Biotechnology, Lake Placid, NY, USA) and 0.05 μg RENILLA luciferase driven by the SV40 promoter (Promega, Madison, WI, USA) were transfected into cells using FuGENE 6 Transfection Reageant (Roche) according to the manufacturer’s protocol. After 24 h transfection, cells were incubated for another 24 h with Wnt3a-CM or L-CM, in the presence of increasing doses of RSV. After the last 24 h of incubation, the cells were lysed and luciferase activity was evaluated using the dual luciferase assay kit (Promega). Values for TOPflash luciferase activity were normalized to those of Renilla activity.

### Sirt1 activity assay in OA Ob

Sirt1 activity was determined in whole cell lysates using the Abcam Sirt1 Activity Assay Kit (Fluorometric, ab156065). Cells treated with RSV for 24 h were harvested with cell lysis buffer under non-denaturing conditions. Briefly, medium was removed and cells were rinsed with ice-cold PBS and then 75 ul of 1X ice-cold cell lysis buffer added to each well and incubated on ice for 5 minutes. Cells were scrapped and transferred and sonicated four times for 5 seconds each on ice and centrifuged for 10 minutes at 4 °C. The supernatant is the whole cell lysate. Determinations were performed in triplicate for each preparation.

### Detection of Sirt1 in human bone tissue by immunohistochemical analysis

Full-thickness specimens from the tibial plateaus were processed for immunohistochemical analysis using the protocol for immunohistochemical paraffin-embedded staining sections (Abcam, ab64264). Briefly, slides were incubated for 10 minutes in 10 mM sodium citrate buffer pH 6.0 at 80 °C and then slides were cooled on the bench top for 30 minutes. Slides were incubated with PBS, 0.4% Triton 1% BSA for 10 minutes at room temperature. The slides were then incubated with hydrogen peroxide block for 10 minutes followed by protein block for another 10 minutes at room temperature. The primary antibodies against Sirt1 (1:100, NBP1-49540) were applied overnight at 4 °C in a humidified chamber. Slides were incubated in the presence of a biotin-conjugated goat anti-polyvalent for 10 minutes at room temperature. This was followed by the addition of the streptavidin peroxidase complex for 10 minutes, and slides were counterstained with eosin. Sections were examined under a light microscope (Leitz Orthoplan; Leica) and photographed using a CoolSNAP cf Photometrics camera (Roper Scientific, USA).

### Statistical analysis

All quantitative data are expressed as mean ± SEM. The data were analyzed by Student’s *t* test when comparing two groups. In experiments comparing three groups, we performed analysis of variance (ANOVA) and the post-hoc Fisher least significant difference (LSD) protected *t* test; *p* values <0.05 were considered statistically significant.

## Results

### Effect of RSV on altered phenotype in OA Ob

**Fig. 1:**
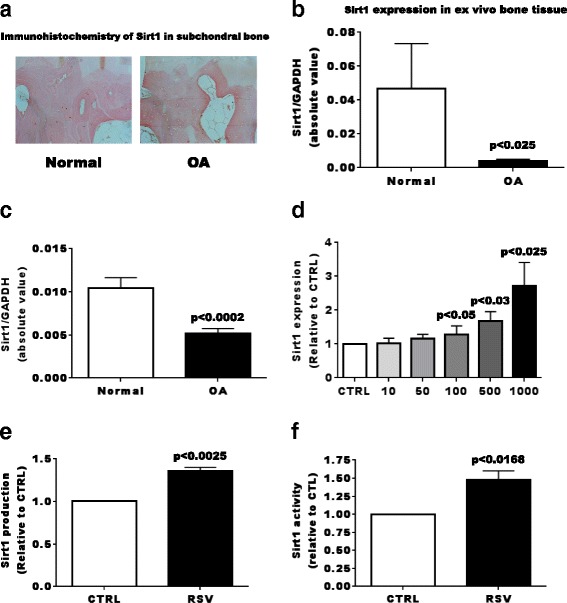
Effect of resveratol (RSV) on sirtuin 1 (*Sirt1*) and P300 levels. **a** Representative immunohistochemical determination of Sirt1 protein expression in normal subchondral bone tissues and in osteoarthritis (*OA*). **b** Sirt1 expression by normal (*n* = 3) and OA (*n* = 6) ex vivo subchondral bone explants. **c** Sirt1 expression by normal (*n* = 9) and OA osteoblasts (*Ob*) (*n* = 21). **d** Confluent OA Ob were treated with increasing doses of RSV for 24 h and lysed in TRIzol^TM^ for qRT-PCR analysis of Sirt1 messenger RNA expression (*n* = 5). **e** Quantification of Sirt1 levels as detected in Fig. 2c. **f** Sirt1 activity analysis in OA Ob cell lysates in response to RSV (*n* = 3). *GAPDH* glyceraldehyde-3-phosphate dehydrogenase, *CTRL* control

**Fig. 2:**
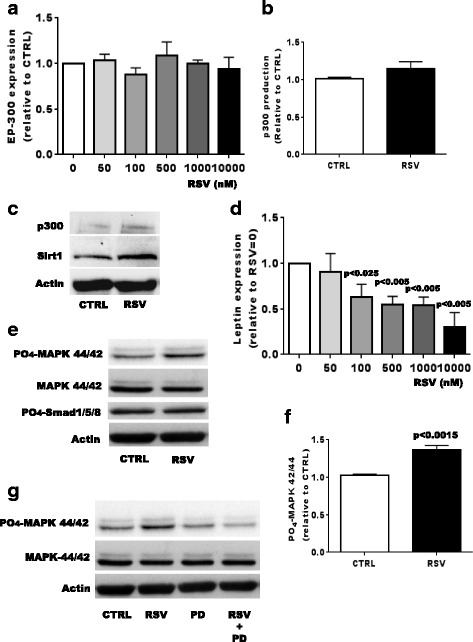
Effect of resveratol (*RSV*) EP-300 and leptin expression, extracellular signal-regulated kinase (Erk)1/2 and Smad1/5/8 signaling activities in osteoarthritis (*OA*) osteoblasts (*Ob*). Confluent OA Ob were treated with increasing doses of RSV for 24 h and lysed in TRIzol^TM^ for qRT-PCR analysis. **a** EP-300 messenger RNA (mRNA) expression (*n* = 6). **b** EP-300 protein abundance in response to 24 h of RSV treatment at 500 nM. **c** Western blot analysis of EP-300 and sirtuin 1 (*Sirt1*) in response to 24 h of RSV treatment at 500 nM. **d** Leptin mRNA expression (*n* = 6). Results are given as the mean value ± SEM of markers relative to glyceraldehyde-3-phosphate dehydrogenase (*GAPDH*). **e** Representative western blot analysis of OA Ob treated or not with 500 nM of RSV for 1 h. Phosphorylated and non-phosphorylated Erk1/2, phosphorylated Smad1/5/8, and β-actin were detected by western blot analysis (*n* = 6 experiments). **f** Quantification of phosphorylated and non-phosphorylated Erk1/2 levels as detected in **e. g** Specific inhibition of RSV-dependent phosphorylated Erk1/2 by PD98059. *CTRL* control, *MAPK* mitogen-activated protein kinase

We first evaluated the expression of Sirt1 in normal and OA subchondral bone tissues. As shown in Fig. 1a, Sirt1 was readily observed in normal bone tissue whereas it was much reduced in OA bone tissue. The expression of Sirt1 in ex vivo bone samples also demonstrated robust expression in normal bone explants, whereas it was significantly reduced in OA explants (Fig. 1b). As we previously reported that Sirt1is reduced in OA Ob and leads to alteration of Ob functions [10], we determined that indeed, OA Ob had reduced Sirt1 expression compared to normal Ob (Fig. 1c). Since RSV is known to stimulate Sirt1 activity, we then attempted to determine if RSV treatments could increase Sirt1 expression and production in OA Ob. Indeed, there was almost threefold dose-dependent stimulation by RSV of Sirt1 expression (Fig. 1d), production (Figs. 1e and 2c) and activity (Fig. 1f) in OA Ob. Whereas RSV can stimulate p300 levels in other cell systems [33], the addition of RSV did not significantly affect p300 levels in OA Ob (Fig. 2a-c).

The expression of leptin, which is already elevated in OA Ob, contributes to their abnormal function as we previously reported [28]. RSV is known to regulate leptin expression in other cell systems [31], hence we tested whether this could also be the case for OA Ob. Indeed, RSV dose-dependently decreased the expression of leptin approximately twofold in OA Ob (Fig. 2d). As OA osteoblasts also have altered responses to insulin-like growth factor 1 and leptin treatments [20], in part due to altered Erk1/2, and since in the present study RSV corrected the expression of leptin, we evaluated the effect of RSV treatments on altered Erk1/2 and other signaling pathways. Our results indicated that RSV increased the phosphorylation of Erk1/2, which is involved in the control of cell proliferation, differentiation and apoptosis (Fig. 2e and f). In contrast, the addition of RSV did not significantly affect the phosphorylation of Smad 1/5/8 levels (Fig. 2e). Using PD 98059 which selectively inhibits the phosphorylation of Erk1/2, we also showed that it prevented the RSV-dependent stimulation of phosphorylated Erk1/2 in OA osteoblasts (Fig. 2g).

**Fig. 3:**
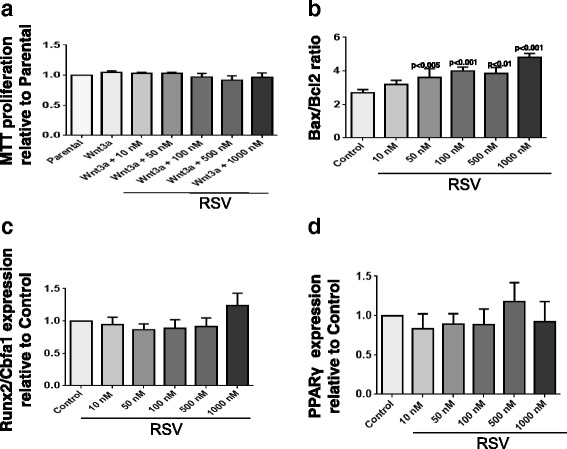
Effect of resveratol (*RSV*) on proliferation, bax/bcl2, runx2/cbfa1, and peroxisome proliferator-activated receptor γ (*PPARγ*) expression in osteoblasts (Ob) in osteoarthritis (OA). **a** OA Ob were plated at 10,000 cells/cm^2^ and allowed to attach overnight in BGJ medium containing 10% FBS. Cells were then treated with the same medium with 0.5% FBS for 24 h prior to receiving increasing doses of RSV (10 , 50 , 100, 500 or 1000 nM) or the vehicle in the same medium for another incubation of 24 h. Cell proliferation was assessed by the MTT assay (*n* = 5). Confluent OA Ob were treated with increasing doses of RSV for 24 h and lysed in TRIzol^TM^ for qRT-PCR analysis. **b** BAX/BCL2 messenger RNA (mRNA) expression (*n* = 6). **c** Runx2/Cbfa1 mRNA expression (*n* = 7). **d** PPARγ mRNA expression (*n* = 6). Results are given as the mean value ± SEM of markers relative to glyceraldehyde-3-phosphate dehydrogenase

As OA Ob grow faster than normal Ob in vitro [20], we determined the importance of RSV in the proliferation and the viability of OA Ob. Our results indicate that RSV had little effect on cell proliferation as assessed by the MTT assay (Fig. 3a) and slightly affected the Bax/Bcl2 ratio, an indicator of cell survival (Fig. 3b). Since the runt-related transcription factor 2 (Runx2/Cbfa1) pathway has been identified as a master regulator of the Ob-specific expression of osteocalcin [34], which is elevated in OA Ob [2], we therefore analyzed the ability of RSV to control the expression of Runx2/Cbfa1. Our results indicated that RSV had no effect on Runx2/Cbfa1 expression (Fig. 3c). Similarly, increasing doses of RSV did not alter the expression of PPARγ (Fig. 3d).

**Fig. 4:**
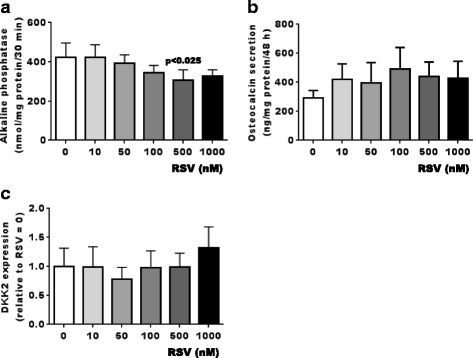
Effect of resveratol (*RSV*) on alkaline phosphatase (ALP) activity, osteocalcin secretion and dickkopft-2 (*DKK2*) expression in osteoblasts (Ob) in osteoarthritis (OA). Confluent OA Ob were treated with increasing doses of RSV (10, 50, 100, 500 or 1000 nM) or the vehicle for 48 h in presence of 1,25(OH)_2_D_3_. Cell culture medium was collected for the determination of osteocalcin secretion and cell lysates were used for the determination of ALP activity. **a** ALP activity in OA (*n* = 6) Ob. **b** Osteocalcin release by OA (*n* = 6) Ob. Confluent OA Ob were treated with increasing doses of RSV for 24 h and lysed in TRIzol^TM^ for qRT-PCR analysis. **c** DKK2 mRNA expression (*n* = 4). Results are given as the mean ± SEM value of markers relative to glyceraldehyde-3-phosphate dehydrogenase

As alkaline phosphatase activity and osteocalcin release are elevated in OA Ob as compared to normal Ob [2], and since recent studies have shown an association between dietary polyphenols and the prevention of OA [35], we tested the effect of RSV on altered ALP activity and osteocalcin release in human OA Ob. Our results showed that RSV at a high dose only of 500 nM reduced ALP in OA Ob (Fig. 4a), indicating that RSV can partially reverse the abnormal ALP activities observed in OA Ob. However, the addition of RSV did not significantly affect osteocalcin release in OA Ob (Fig. 4b). Under similar conditions, RSV treatments did not affect the gene expression of ALP and OC (data not illustrated).

#### Effect of RSV on mineralization in OA Ob

**Fig. 5:**
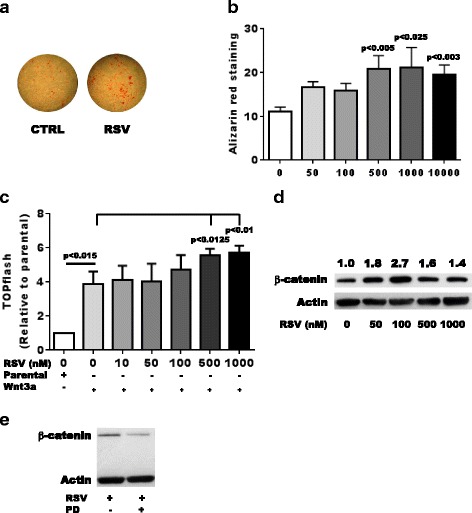
Effect of resveratol (*RSV*) on Wnt/β-catenin signaling activity and mineralization in osteoblasts (Ob) in osteoarthritis (OA). OA osteoblasts were stimulated with increasing doses of RSV (10, 50, 100, 500 or 1000 nM) for up to 28 days. **a** Representative alizarin red staining of normal Ob. **b** Quantification of alizarin red staining as a function of time and chronic effect of RSV exposure in OA Ob (*n* = 6). Cells were treated for 4 h with vehicle or increasing doses of RSV. **c** TOPflash activity in OA Ob in response to parental and Wnt3a conditioned medium either in the presence or absence of RSV for 24 h. Values are reported relative to values in parental samples (*n* = 5 experiments). **d** Western blot analysis of β-catenin expression in OA Ob in response to Wnt3a in the presence of RSV. Representative of four experiments. **e** Western blot analysis of the effect of PD98059 on RSV-dependent β-catenin activation in OA Ob. Representative of three experiments. *CTRL* control

The mineralization of OA Ob is reduced compared to normal Ob, as we previously reported [2], and reflects the in vivo situation [5, 36]. The reduction in in vitro mineralization was due to an increase in the endogenous production of TGF-β1 by OA Ob, which could be reduced by stimulating Sirt1 activity in OA Ob with β-nicotinamide mononucleotide (NMN) [10]. Of note, Sirt1 is expressed in bone cells and promotes bone formation [37] and on the other hand it reduces osteoclastogenesis [38]. Osteoblast deletion of Sirt1 in mice leads to delayed bone mineralization [39]. As RSV stimulates Sirt1 activity [10] and we showed herein that the expression, production and activity of Sirt1 were increased in OA Ob following RSV treatment, we tested whether RSV could play a role on OA Ob mineralization. As shown in Fig. 5a and b, increasing doses of RSV increased the mineralization potential of OA Ob about twofold as assessed by alizarin red staining.

#### Effect of RSV on DKK2 expression in OA Ob

We previously reported that elevated DKK2 levels are responsible for the elevated ALP in OA Ob and that correcting DKK2 levels by small interfering RNA (siRNA) techniques reduced the level of ALP in OA Ob to that in normal Ob [9]. Since RSV partly corrected the altered elevated ALP, we tested whether RSV could have an effect on the expression of the Wnt antagonist DKK2. Our results indicated that increasing doses of RSV had no effect on DKK2 expression (Fig. 4c), suggesting that RSV acts differently on ALP.

#### Role of RSV on altered Wnt/β-catenin signaling

We previously reported that OA Ob have an altered Wnt/β-catenin signaling pathway [9], hence we questioned whether RSV treatment could correct this activity. When OA Ob were treated with Wnt3a-CM, the canonical Wnt signaling pathway was increased about fourfold (Fig. 5c). The presence of increasing doses of RSV further stimulated this activity up to sixfold (Fig. 5c). This increase in Wnt/β-catenin signaling activity was accompanied by a significant increase in free β-catenin levels measured by immunoblotting assays (Fig. 5d). We next evaluated what triggered this increase in β-catenin activity in response to RSV. Using the selective Erk1/2 inhibitor PD98059, which reduced Erk1/2 phosphorylation in OA Ob (Fig. 2g), we observed that it also reduced the RSV-dependent β-catenin activation observed in these cells (Fig. 5e). Indeed, PD98059 reduced RSV-dependent β-catenin levels by 36.1 ± 10.5%.

## Discussion

Resveratrol, a natural polyphenol is known for its anti-inflammatory, anti-oxidant, anti-aging, anti-carcinogenic, cardioprotective and neuroprotective properties [40–42]. The protective effect of RSV on articular cartilage degradation was first reported by Elmali et al. in a rabbit model of OA [43]. In human articular chondrocytes, RSV has an anti-apoptotic and anti-inflammatory effects [44, 45]. Similarly, Im et al. demonstrated potent anabolic and anti-catabolic potential of RSV in human adult articular chondrocytes via inhibition of matrix-degrading enzyme [46]. Taken together, these findings suggest that RSV may protect against cartilage degeneration and have protective effects against OA. RSV enhances osteoblast activities in bone tissue, and stimulates Ob proliferation and differentiation and therefore promotes bone formation and further suggests a future role for RSV in fracture healing [14]. However, the role of RSV on altered subchondral bone has not yet been elucidated and since the subchondral bone plays a critical role in the process of the initiation and/or the progression of OA, it is necessary to address whether RSV can play a beneficial role in altered subchondral bone in patients with OA.

In the present study, we showed the beneficial effect of RSV on the altered phenotype of human OA Ob and the mechanism by which RSV acts. We previously reported that Sirt1 expression was significantly reduced in OA Ob compared to normal Ob and is responsible, at least in part, for the increased expression of TGF-β1 and SOST, which can both alter the phenotype of human OA Ob [10]. Herein, we demonstrated that Sirt1 expression is reduced in OA subchondral bone tissue, ex vivo explants, and in vitro Ob. Stimulating Sirt1 activity with β-nicotinamide mononucleotide reduced the elevated expression of TGF-β1 and SOST in OA Ob, and corrected the phenotype and altered mineralization of OA Ob [10]. Of note, TGF-β1 is upregulated by leptin, which is elevated in OA Ob and contributes to the abnormal function of these cells as we showed previously [20]. We first demonstrated that RSV increased Sirt1 expression, production and activity in OA Ob. This upregulation of Sirt1 observed in OA Ob following treatment with RSV is consistent with previous studies in which RSV was identified as an activator of Sirt1 in OA chondrocytes [47]. Second, the expression of leptin, which is elevated in OA Ob and contributes to their abnormal function, was decreased in OA Ob when treated with RSV. Taken together, these results and our previously published data indicate that leptin and TGF-β1 are downstream targets of RSV via its stimulation of Sirt1 activity in OA Ob, a situation that could link reduced Sirt1 levels in OA Ob with a number of abnormal biomarkers in these cells. However, in contrast to previously reported studies [33], we did not establish a link between EP-300 and Sirt1 activation following RSV stimulation in OA Ob.

We previously demonstrated that leptin alters a number of intracellular cell signaling pathways, namely Erk1/2, in OA osteoblasts [20]. Since stimulating SIRT1 activity with RSV reduced the elevated expression of leptin in OA Ob, we therefore questioned whether this could correct the abnormal phenotype and altered mineralization observed in OA Ob. Our study revealed that RSV has an effect on the altered intracellular Erk1/2 signaling pathway, while it was without effect on the Smad1/5/8 signaling pathway in OA Ob. Indeed, RSV treatments increased the phosphorylation of Erk1/2, which is involved in the control of cell proliferation, differentiation and apoptosis.

It has been demonstrated by Li et al. that RSV inhibits proliferation and promotes apoptosis of osteosarcoma cells [16]. As OA Ob proliferate faster than normal Ob, we determined the importance of RSV in the proliferation and the viability of OA Ob cells. Our results indicated that RSV had little effect on cell proliferation and slightly affected the Bax/Bcl2 ratio, an indicator of cell survival. These results indicate that RSV could not correct the abnormal proliferation of human OA Ob. However it has been shown that RSV induced the proliferation and differentiation of human bone marrow-derived mesenchymal stem cells (MSC) [48] and of the Ob precursor cell line derived from mouse calvaria, MC-3 T3 cells [15]. As OA Ob proliferate faster and are more differentiated than human bone marrow-derived MSC and the Ob precursor MC-3 T3 cells, this would explain again that RSV has a different role to play in well-differentiated cells vs pre-osteoblasts, hence that its effect depends on the state of differentiation and the type of cells.

The Runx2 pathway has been identified as a master regulator of Ob-specific expression of OC, which is elevated in OA Ob [37]. We therefore analyzed the ability of RSV to control the Runx2 and PPARγ in OA Ob. Our results indicate that RSV had no effect on Runx2 expression and on PPARγ. These results confirmed that RSV could not regulate Runx2 and therefore it was expected that it would not correct the abnormal OC release in OA Ob. However, RSV partially corrected the abnormal ALP activities in OA Ob, whereas the addition of RSV did not significantly affect elevated OC release in OA Ob. In contrast, RSV promotes the activity of ALP in rat bone marrow-derived MSC [47], indicating again that RSV has a different role depending on the context and the type of cells.

Previous reports from our laboratory [2, 10, 26] and other investigators [4, 5] have shown a reduction in mineralization in OA Ob and in OA bone tissue, respectively, and reflects the in vivo situation. The reduction in in vitro mineralization was due to an increase in TGF-β1, which was slightly reduced by stimulating Sirt1 activity in OA Ob with NMN. As RSV stimulate Sirt1 activity, we tested whether RSV could play a role on OA Ob mineralization. We showed that increasing doses of RSV in OA Ob increased their mineralization potential about twofold as assessed by alizarin red staining.

Zhou et al. demonstrated that RSV elevates the expression of β-catenin in the early stages of MSC differentiation and that knockdown of *SIRT1* inhibits Wnt/β-catenin signaling, while RSV treatment or overexpression of *SIRT1* activates Wnt/β-catenin signaling [49]. Indeed, in the present study, we observed that RSV increased the Wnt3a-dependent Wnt/β-catenin activity in OA Ob using the dual TOPflash/Renilla reporter assay. This increase in Wnt/β-catenin signaling was accompanied by an increase in free β-catenin levels measured by immunoblotting assays in OA Ob. We previously reported that the abnormal expression of phenotypic markers and reduced mineralization of OA Ob are linked with the stimulation of the Wnt antagonist DKK2 [9] and SOST [10] and the inhibition of the Wnt agonist, R-spondin 2 [11]. Since RSV partly corrected the altered elevated ALP, yet increased abnormal Wnt signaling and altered mineralization in human OA Ob, we tested whether RSV could have an effect on the elevated expression of the Wnt antagonist DKK2 in OA Ob, which is responsible in part for the altered ALP in these cells. Our results indicated that increased dose response to RSV had no effect on DKK2, suggesting that RSV acts differently on ALP activity bypassing DKK2. Mak et al. showed that the antagonist DKK1 inhibits Wnt3a-induced β-catenin in MSC cells and that RSV abolishes this inhibitory effect [50]. Moreover, they showed that RSV increases Wnt signaling by reducing the level of glycogen synthase kinase 3β (GSK-3β), which phosphorylates and destabilizes β-catenin, and that phosphorylation of GSK-3β requires Erk1/2. Indeed our result indicated that RSV increases the phosphorylation of ERK1/2 and increases Wnt signaling in OA Ob.

## Conclusion

These data indicate that RSV inhibits the endogenous expression of leptin by OA osteoblasts and promotes the Wnt/β-catenin and Erk1/2 signaling pathways, which are altered in these cells. This last situation could explain the role of RSV in the in vitro mineralization, which is altered in these cells. These data suggest the potential role of RSV in OA.

## Abbreviations

1,25(OH)_2_D_3_: Active form of vitamin D_3_
ALP: Alkaline phosphatase
BSA: Bovine serum albumin
cWnt: Canonical Wnt/β-catenin signalling
DKK2: Dickkopft-2
DMEM: Dulbecco’s modified Eagle’s medium
EP300: E1A binding protein p300
Erk: Extracellular signal-regulated kinase
FBS: Fetal bovine serum
GAPDH: Glyceraldehyde-3-phosphate dehydrogenase
MAPK: Mitogen-activated protein kinase
MSC: Mesenchymal stem cells
NMN: β-Nicotinamide mononucleotide
OA: Osteoarthritis
Ob: Osteoblasts
OC: Osteocalcin
OM: Osteogenic induction medium
PPARγ: Peroxisome proliferator-activated receptor γ
qRT-PCR: Quantitative reverse transcriptase-polymerase chain reaction
RSV: Resveratol
Runx2: Since runt-related transcription factor 2
SIRT1: Sirtuin 1
SOST: Sclerostin
TFG-β1: Transforming growth factor β-1
TOPflash: TCF/Lef luciferase assay
WB: Western blot
